# Prosocial Behaviors Following Mortality Salience: The Role of Global-Local Identity

**DOI:** 10.3390/bs15111494

**Published:** 2025-11-03

**Authors:** Bo Chen

**Affiliations:** Graduate School of China, Sungkyunkwan University, Seoul 03063, Republic of Korea; chenbo@skku.edu

**Keywords:** mortality salience, prosocial behaviors, global-local identity, social connectedness

## Abstract

This research examines how reminders of mortality influence prosocial behavior through the lens of terror management theory. We propose that these effects depend on individuals’ global–local identity—the degree to which they identify with the broader world versus a local community. In two experimental studies, participants were exposed to mortality salience manipulations and then reported their intentions to engage in prosocial behaviors. The results consistently showed that mortality salience increased prosocial intentions for individuals with a global identity but not for those with a local identity. This interaction was explained by differences in perceived social connectedness. Together, these findings highlight the role of global–local identity in shaping prosocial responses to mortality reminders, offering theoretical insights into terror management processes and practical implications for fostering prosociality in diverse social contexts.

## 1. Introduction

From the global pandemic to ongoing wars, recent years have witnessed a surge in death-related anxiety, fueled by a rapidly changing and highly uncertain world. This heightened sense of anxiety has profoundly shaped people’s thoughts and behaviors (Bergman, 2024). According to Terror Management Theory (TMT), human beings possess an inherent tendency to avoid contemplating death and to seek mechanisms that reduce death-related anxiety (Burke et al., 2010; Greenberg et al., 1994). A substantial body of research has shown that when mortality salience increases, individuals tend to pursue psychological buffers to cope with the resulting existential threat (e.g., Li et al., 2025; Pyszczynski et al., 2004). However, recent studies have raised important concerns about the robustness and replicability of TMT effects (e.g., L. Chen et al., 2025). These debates highlight the need to move beyond assuming that mortality salience produces uniform outcomes and instead to identify the boundary conditions that determine when and why individuals engage in particular responses.

One such response is engaging in prosocial behaviors, such as charitable giving (e.g., Cai & Wyer, 2015) and ethical consumption (e.g., Cheng et al., 2024). Because these actions align with shared societal standards and norms, they enable individuals to reaffirm their worldview and bolster self-esteem, thereby alleviating existential fears and fostering a sense of psychological security (Solomon et al., 1991). However, evidence linking mortality salience to prosociality remains mixed. Some studies have reported self-focused or defensive reactions instead (Jonas et al., 2002; Hirschberger et al., 2008). Prior research suggests that these divergent outcomes may hinge on both contextual and individual-level factors, such as cultural background, the type of helping behavior, and the nature of the beneficiaries. Building on this line of inquiry, the present research examines how global versus local identity functions as a critical moderator of prosocial responses to mortality salience.

Although numerous studies have shown that individuals often engage in prosocial behaviors in response to mortality salience (e.g., Cheng et al., 2024; Jin & Ryu, 2022), research examining individual differences in these behaviors remains limited (e.g., Li & Guan, 2024). Self-identity plays a crucial role in shaping how people cope with death-related thoughts elicited by their environment (Landau et al., 2009). In particular, in today’s globalized world, individuals’ global versus local identity influences how they relate to the broader world (Arnett, 2002). A person’s global-local identity can orient attention either toward global issues or toward one’s local community, thereby shaping perceptions of social connectedness and guiding behavioral orientations (e.g., Zhang & Khare, 2009; Salnikova et al., 2022). Accordingly, we propose that global-local identity moderates individuals’ prosocial responses when confronted with mortality-related threats. Overall, the present research investigates how mortality salience interacts with global-local identity to influence individuals’ intentions to engage in prosocial behavior.

## 2. Theoretical Background

### 2.1. Mortality Salience and Prosocial Behavior

The Terror Management Theory (TMT) (Greenberg et al., 1986, 1994) posits that although individuals are driven by an innate desire to preserve life, they are also uniquely aware of their inevitable death. When reminded of death, a state known as mortality salience, such as through exposure to information about fatal accidents or war casualties, individuals experience heightened anxiety and threat. In response, they activate psychological defense mechanisms to mitigate this existential anxiety (Greenberg et al., 1994). Prior research has proposed a dual-component anxiety buffer, consisting of adherence to cultural worldviews and enhancement of self-esteem (Jonas et al., 2002; Pyszczynski et al., 1999). By aligning with widely shared beliefs or worldviews, individuals achieve a sense of safety and meaning, thereby reducing anxiety (Wisman et al., 2015). Empirical findings further suggest that under mortality salience, individuals reinforce their ingroup ties to secure social connectedness, which in turn supports worldview defense (e.g., Routledge et al., 2004). In addition to worldview affirmation, mortality salience can also lead individuals to engage in self-focused behaviors that enhance self-esteem, such as indulging in hedonic consumption or acquiring short-term material possessions (e.g., Guan et al., 2015).

Based on TMT, researchers have examined various strategies individuals use to buffer death-related fear or anxiety (e.g., Guan et al., 2015; Jonas et al., 2002). While earlier studies suggest that mortality salience may lead individuals to adopt more self-focused behaviors in an effort to bolster self-esteem (e.g., Chopik & Edelstein, 2014; Fransen et al., 2008), a growing body of research indicates that mortality salience can also promote prosocial behaviors (e.g., Cai & Wyer, 2015; Cheng et al., 2024; Dunn et al., 2020). For instance, Ferraro et al. (2005) found that mortality salience enhanced participants’ intention to donate to charity, notably among consumers who already valued virtuous behavior. In many cultures, societal norms regard benevolence and concern for others as highly valued personality traits (Jonas et al., 2002). According to TMT, because prosocial behaviors are endorsed by cultural worldviews, engaging in activities that support and benefit others can serve as a psychological defense against the anxiety and fear triggered by death-related thoughts.

### 2.2. The Moderating Effect of Global-Local Identity

While prior literature has highlighted the influence of mortality salience on individuals’ thoughts and behaviors (e.g., Li et al., 2025), relatively few studies have examined how individual identity shapes actions taken to alleviate death-related anxiety, particularly prosocial behaviors. Because prosocial behaviors involve actions that benefit the well-being of others, identity, which shapes how individuals relate to others and to the broader world, is likely a key factor influencing these behaviors. Arnett (2002) proposes that individuals in modern societies possess both local and global identity characteristics. A global identity fosters a sense of connection with people across the world, whereas a local identity emphasizes affiliation with one’s immediate community (Zhang & Khare, 2009). Individuals with a salient global identity tend to report higher levels of perceived social connectedness than those with a local identity, as they often feel connected to a broader set of people, including unfamiliar individuals from distant places (Pong & Tam, 2023). Moreover, although global and local identities can be considered relatively stable traits, research has shown that they can also be temporarily influenced by situational cues such as priming tasks (e.g., Gao et al., 2017; Salnikova et al., 2022).

We propose that individuals’ global or local identity moderates the effect of mortality salience on their prosocial behavior. When a global identity is active, individuals tend to view themselves connected to a unified world and are thus more likely to express concerns for the wellbeing of people on a global scale (e.g., Arnett, 2002; Grinstein & Riefler, 2015). This tendency is likely to be amplified in the presence of mortality salience. Under the influence of mortality salience, the social value of prosocial behavior aligns with the cultural worldview of people with a global identity. Consequently, these individuals are more likely to engage in prosocial actions as a way to seek social support and alleviate death-related anxiety (Cheng et al., 2024). In contrast, individuals with a local identity are more focused on their limited community and are less concerned with the lives of distant others (Ng & Basu, 2019). Since people feel less connected to the broader world, prosocial activities are less likely to serve as an effective buffer against anxiety caused by mortality salience. In sum, we propose that the positive effect of mortality salience on individuals’ prosocial behavior will be moderated by their identity, such that this positive effect will be more pronounced for individuals with a global identity than for those with a local identity.

### 2.3. The Mediating Role of Perceived Social Connectedness

Previous research has identified social connectedness as a key mechanism through which human beings seek existential meaning (e.g., Deci & Ryan, 1987; Routledge et al., 2004). According to Terror Management Theory (TMT), when individuals experience anxiety activated by mortality salience, they tend to affirm their cultural worldviews and conform to societal standards (e.g., Routledge et al., 2004; Wisman & Koole, 2003). This process can lead individuals to perceive a higher level of social connectedness with others and the broader world (Mikulincer et al., 2003), particularly among those who hold a global rather than a local identity.

Perceived social connectedness refers to an individual’s generalized sense of being interconnected with others and the broader social world, extending beyond immediate groups or communities (Lee & Robbins, 1995; Mikulincer et al., 2003). This generalized connectedness serves as a mechanism through which mortality salience influences prosocial behavior, particularly among individuals with a global identity. By focusing on this construct, we capture the mechanism through which mortality salience and global-local identity influence prosocial behavior, providing individuals with relational support that buffers death-related anxiety. As a result, individuals experiencing heightened social connectedness may become more motivated to engage in prosocial behaviors as a way to manage mortality-related anxiety. Therefore, we propose that perceived social connectedness mediates the interactive effect of mortality salience and global-local identity on individuals’ tendencies to engage in prosocial behavior.

### 2.4. The Current Study

**Figure 1:**
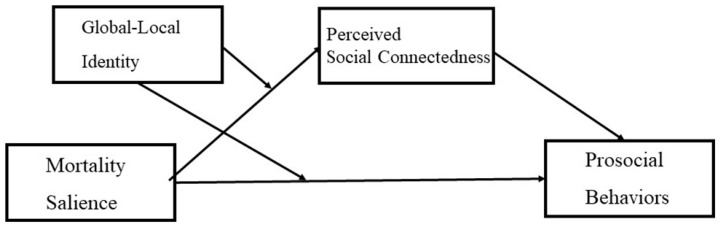
Theoretical Framework.

This research aims to investigate the joint effect of mortality salience and people’s global-local identity on prosocial behavior as well as the underlying mechanism of perceived social connectedness. The theoretical framework is summarized in Figure 1. Two experiments were conducted to test the proposed effects. Specifically, we examined two forms of prosocial behaviors under mortality salience as dependent measures, namely ethical consumption in Study 1 (e.g., Bray et al., 2011) and charity donation in Study 2 (e.g., Dunn et al., 2020). In Study 1, participants’ chronic global–local identity was measured, whereas in Study 2, it was experimentally manipulated.

## 3. Methodology

### 3.1. Study 1

This study employed a 2 (*Mortality salience “MS”* versus *Control*) × 2 (*Global Identity* versus *Local Identity*) between-subjects online experiment, in which mortality salience was manipulated and global–local identity was measured.

#### 3.1.1. Participants

G*Power (version 3.1.9.7) indicated that at least 128 participants were needed to discern differences with 80% power with an effect size of 0.25 and an α level of 0.05. We recruited 175 participants from Mturk. We measured their gender, age, education level, and income. Gender was treated as a dummy variable (54% male). Age was measured in actual number (M_age_ = 25, SD = 2.3). Education level was measured with five categories (year nine and below, high school, college diploma, bachelor’s degree, postgraduate degree and above). We measured income level with an ordinal variable including seven categories.

#### 3.1.2. Manipulations

Participants were randomly assigned to either the mortality salience (“MS”) condition or the control condition. Following prior studies (e.g., Mandel & Smeesters, 2008), participants in the MS condition were exposed to a news report on the death toll during the COVID-19 pandemic, and then they were asked to write down their thoughts about it. In the control condition, participants were asked to write down their thoughts after visiting the dentist. Since previous research suggests that the effect of MS manipulation on the dependent variable becomes more accessible and effective when a delay occurs between the manipulation and the dependent variable measurement (Pyszczynski et al., 1999), we gave participants a filler task right after the manipulation, which concerns a product evaluation.

After measuring the dependent variable, participants completed a 19-item scale assessing their global–local identity (Arnett, 2002; Zhang & Khare, 2009). Nine items assessed global identity, and ten items assessed local identity; responses were averaged to create separate composite scores for each dimension. Participants whose local score exceeded their global score were categorized as having a local identity, whereas those whose global score exceeded their local score were categorized as having a global identity, reflecting their chronic identity (Zhang & Khare, 2009). In total, 99 participants were classified as having a global identity, and 76 participants were classified as having a local identity.

#### 3.1.3. Measures

We measured participants’ ethical consumption intentions using a seven-item, seven-point Likert scale, adopted from Y. Chen and Moosmayer (2020) and Cheng et al. (2024) and based on the Socially Responsible Purchase and Disposal Scale (Mohr & Webb, 2005). An example item is “I will not buy from companies that harm animals or plants.” The scale showed good reliability (α = 0.88). Participants’ gender, age, education, and income were recorded as potential covariates, but including them did not change the results, so we report ANOVA for simplicity.

#### 3.1.4. Results

**Figure 2:**
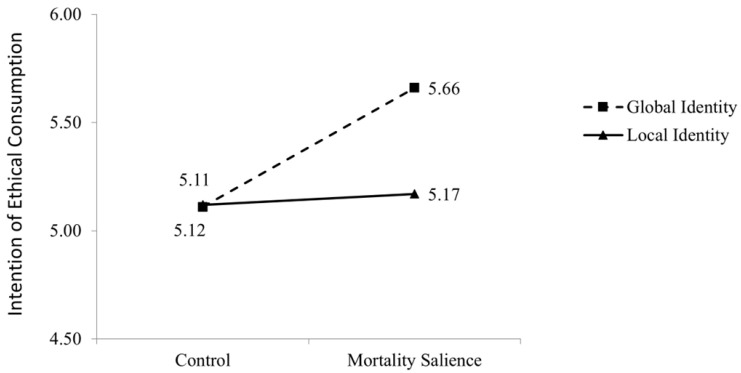
Results of Study 1.

We conducted an ANOVA with mortality salience and participants’ global-local identity as independent variables, and participants’ ethical consumption intentions as the dependent variable. Results revealed a significant main effect of mortality salience (F(1, 174) = 4.5, *p* = 0.03), that is, participants in the mortality salience condition showed a higher intention of ethical consumption than those in the control condition (M_mortality_ = 5.42 versus M_control_ = 5.14; t(173) = 2.41, *p* = 0.03). The main effect of participants’ global-local identity was also significant (F(1, 174) = 4.19, *p* = 0.04). In general, participants with a global identity are more likely to consume ethically than those with a local identity (M_global_ = 5.39 versus M_local_ = 5.13; t(173) = 2.41, *p* = 0.02). The interaction effect between the two factors was also significant (F(1, 174) = 7.1, *p* < 0.01). Follow-up contrast analyses showed that, for participants with a global identity, mortality salience exerted a positive effect on their ethical consumption intention (M_mortality_ = 5.66 versus M_control_ = 5.11; t(97) = −3.97, *p* < 0.01), but no effect was found among those with a local identity (M_mortality_ = 5.17 versus M_control_ = 5.12; t(74) = 0.37, *p* = 0.76). These results are consistent with our research propositions. Figure 2 visualizes the nature of the interaction effect.

#### 3.1.5. Discussion

Results of Study 1 provided initial support for the moderation effect of individuals’ global–local identity in the relationship between mortality salience and people’s prosocial behaviors. In particular, we found that a local identity attenuated the positive effect of mortality salience on individuals’ ethical consumption intention. It is worth noting that the prosocial behavior examined in this study, ethical consumption, reflects a global cause (i.e., climate and environmental concerns). This focus aligns with our interest in understanding how global–local identity shapes responses to mortality salience in globally relevant contexts.

To enhance the robustness of the findings, we conducted Study 2, which concerned a different type of prosocial behavior, namely charitable donation. In addition, although a person’s global–local identity is generally considered a stable personality trait (Arnett, 2002), substantial research suggests that it can also be situationally activated (e.g., Gao et al., 2020). Therefore, instead of measuring participants’ global–local identity, we manipulated it using a priming task.

### 3.2. Study 2

Study 2 used the same 2 (*Mortality salience “MS”* versus *Control*) × 2 (*Global Identity* versus *Local Identity*) between-subjects design as in Study 1, with the key difference that participants’ global–local identity was experimentally manipulated.

#### 3.2.1. Participants

G*Power software indicated that at least 128 participants were needed to discern differences with 80% power with an effect size of 0.25 and an α level of 0.05. We recruited 191 participants from Mturk. Similar to Study 1, we measured participants’ gender, age, education level, and income. These variables were not included as covariates in the main analyses; however, additional robustness checks controlling for them yielded the same pattern of results.

#### 3.2.2. Manipulations

In Study 1, we manipulated mortality salience by exposing participants to death tolls in the COVID-19 pandemic, which primarily concerns death of others. Following previous research, we altered the manipulation by asking participants to reflect on their own death (e.g., Guan et al., 2015). Participants were asked to indicate whether they agree with a set of ten statements. An example question in the mortality salience condition is “I feel suffering that I cannot escape from death”. In the control condition, the statements concern general negative emotion, i.e., “feel anxious about my future life”.

To manipulate participants’ global–local identity, we adopted the priming task developed by Gao et al. (2017). In the global (local) identity condition, participants were asked to support a “Think Global Movement,” which featured global (local) businesses and focused on global (local) news and cultures from different parts of the world (local community). They were asked to leave their initials to signify their support. A three-item scale (i.e., *At this moment, I feel that: 1*, a local citizen…7, a global citizen) was used after the priming task to check the effectiveness of the manipulation. Manipulation check results showed participants in the global identity condition are more likely to perceive themselves as a global citizen than those in the local identity condition (M_global_ = 5.78 versus M_local_ = 4.13; t(189) = 17.41, *p* < 0.01), thus our manipulation was successful.

#### 3.2.3. Measures

After the manipulation tasks, participants were exposed to a description of a UNESCO program that aims for helping children from developing countries, then they were asked to indicate their intention to donate to this program. Following previous research, we used a seven-point four-item scale to measure participants’ donation intention (Raganathan & Henley, 2008; Urbonavicius et al., 2019). An example item is “I would like to donate to this program”, and the Cronbach’s alpha for this scale is 0.91.

We measured the mediator, namely perceived social connectedness by using an eight-item seven-point Likert Scale (Lee & Robbins, 1995). An example item is “I have no sense of togetherness with my peers”, and the Cronbach’s alpha for this scale is 0.93.

#### 3.2.4. Results

**Figure 3:**
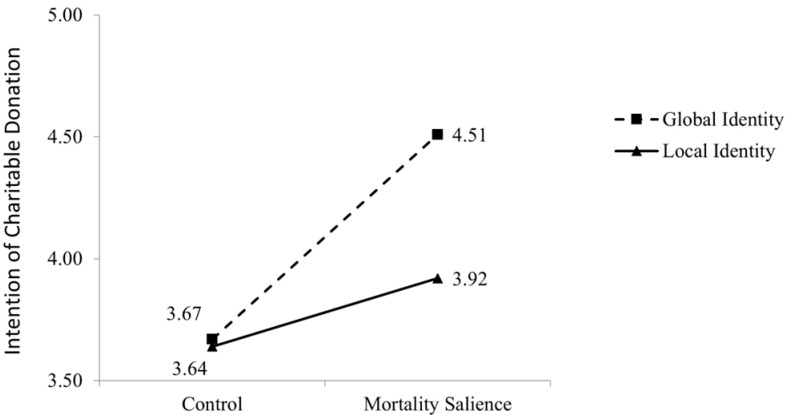
Results of Study 2.

A two-way ANOVA analyses with mortality salience and participants’ global-local identity as independent variables on their intention to donate showed a significant main effect of the mortality salience (F(1, 190) = 19.72, *p* < 0.01) and a significant main effect of the global-local identity (F(1, 190) = 6.06, *p* = 0.02). The interaction effect was also significant (F(1, 190) = 4.69, *p* = 0.03), and the pattern was similar to Study 1. Specifically, when a global identity was primed, participants in the mortality salience condition demonstrated a higher intention to donate to charity than those in the control condition (M_mortality_ = 4.51 versus M_control_ = 3.67; t(94) = −4.76, *p* < 0.01), however, no such effect was found between the two conditions when participants were primed with a local identity (M_mortality_ = 3.92 versus M_control_ = 3.64; t(93) = 1.58, *p* = 0.12). The pattern of the interaction effect was illustrated in Figure 3. Hence, the results of Study 2 corroborate those of Study 1, which is in line with our propositions.

For the mediator, perceived social connectedness, a 2 × 2 ANOVA also revealed a significant interaction between mortality salience and global–local identity (F(1, 190) = 25.39, *p* < 0.01), indicating that the effect of mortality salience on social connectedness depends on identity priming. We then conducted a moderated mediation analysis using Model 8 in PROCESS macro (Hayes, 2018) using mortality salience as the independent variable, global-local identity as the moderator, perceived social connectedness as the mediator, and participants’ donation intention as the dependable variable. The results showed a significant overall indirect effect of the abovementioned interaction effect on participants’ donation tendencies via their perceived social connectedness (indirect effect = −0.35, SE = 0.12, 95% CI: −0.63 to −0.15). Specifically, in the global identity condition, the indirect effect of mortality salience on participants’ intention to donate due to their perceived social connectedness was positive and significant (indirect effect = 0.28, SE = 0.09, 95% CI: 0.23 to 0.48). In contrast, in the local identity condition, the indirect effect of perceived social connectedness was not significant (indirect effect = −0.07, SE = 0.06, 95% CI: −0.20 to 0.02). Therefore, the findings demonstrate that perceived social connectedness mediates the interaction effect between mortality salience and global-local identity on participants’ tendency to donate, which provides support for our proposition.

#### 3.2.5. Discussion

Results of Study 2 offered additional evidence for the moderation role of global–local identity on the effects of mortality salience on individuals’ intentions to engage in prosocial behavior. Our findings held across both participants’ chronic identity and situationally induced identity. Furthermore, we showed that people’s perceived social connectedness serves as the underlying mechanism through which mortality salience promotes prosocial behaviors. Similar to Study 1, the prosocial behavior examined here, donations to UNESCO, reflects a global beneficiary context. This further underscores that our results primarily capture globally oriented prosocial actions rather than prosociality in general.

## 4. General Discussion

### 4.1. Key Results and Theoretical Contributions

Prior research on Terror Management Theory (TMT) has shown that prosocial behavior often functions as a buffer against mortality-induced anxiety (Cai & Wyer, 2015; Cheng et al., 2024). Our research extends this work by demonstrating that such responses are not uniform but depend on individuals’ global–local identity. Across two studies, mortality salience increased prosocial behavior only among those with a global identity. Because the prosocial behaviors examined (ethical consumption and international charitable giving) were tied to global causes, our findings speak most directly to globally oriented prosociality rather than prosocial behavior in general.

This finding advances the dual-component anxiety buffer theory (Pyszczynski et al., 1999) by identifying a boundary condition under which prosocial behavior may not serve as an effective buffer. Specifically, individuals with a local identity appear less inclined to translate mortality concerns into prosocial action. This pattern may reflect the narrower scope of concern inherent in local identity. When mortality is salient, local identifiers are likely to reinforce in-group attachments or cultural norms as coping mechanisms, rather than extend prosociality to a broader collective. In contrast, global identifiers, who define themselves in more inclusive and interconnected terms, are more likely to respond with prosocial behavior, as it aligns with their broader self-construal and provides an effective buffer against existential threat.

Our research also sheds light on the underlying psychological mechanism. We found that perceived social connectedness mediates the effect of mortality salience on prosocial behavior, particularly for individuals with a global identity. This suggests that an existential threat can heighten feelings of connection to humanity at large, which in turn motivates benevolent action. These results highlight perceived social connectedness as a key pathway through which mortality salience translates into prosocial outcomes.

Finally, this work contributes to research on global–local identity (Arnett, 2002; Zhang & Khare, 2009). By both measuring chronic identity and manipulating situational identity, we show that global–local identity meaningfully shapes responses to existential threat. In today’s globalized world, where individuals constantly navigate between local and global identifications, our findings underscore the importance of identity scope in predicting prosocial tendencies.

### 4.2. Practical Implications

This research offers important practical implications for designing interventions to promote prosocial behavior. Our findings help public service administrators better understand people’s thoughts and behavioral tendencies in situations involving death-related threats. While mortality salience can enhance prosocial responses, prior work suggests that messages that evoke excessively high fear may reduce effectiveness (e.g., Witte & Allen, 2000). Therefore, communications should balance mortality reminders with actionable guidance, emphasizing how individuals can make a positive difference. For example, in medical emergencies, messaging that highlights the urgency of blood donations—without inducing overwhelming fear—may increase willingness to help.

These insights are particularly relevant in a post-pandemic context, where reminders of mortality have become more frequent and salient (Nagar & Sharma, 2022; Tao et al., 2021). Messaging that highlights shared vulnerability can strengthen prosocial responses such as volunteering, health compliance, or resource sharing during crises. However, it is important to note that our data examined globally oriented prosocial behaviors. Extensions to local beneficiaries or close ingroup contexts require further empirical validation.

Another key takeaway from our study is the role of global versus local identity in shaping prosocial behavior. People with a salient global identity, those who see themselves as part of a larger collective, are more inclined to act in ways that benefit broader society. Public officials and educators can use this insight to craft messaging and programs that emphasize shared humanity and global interconnectedness. For instance, campaigns addressing climate change, humanitarian aid, or pandemic preparedness can frame these as global challenges requiring collective action, thereby enhancing engagement in globally oriented ethical and cooperative behaviors.

### 4.3. Limitations and Future Research

There are several limitations to the present research that suggest directions for future investigation. First, our study relied on self-reported prosocial tendencies rather than actual behavior. While self-reports provide insight into intentions, they may not fully capture real-world actions due to the well-documented intention–behavior gap (e.g., Carrington et al., 2014). Future research could incorporate behavioral measures, such as actual donations, volunteering, or other incentivized prosocial acts, to enhance external validity and directly test the mechanisms identified in our studies.

Second, we focused on a single identity dimension (global vs. local) and one mediating mechanism (perceived social connectedness). Future research could examine additional factors that may influence prosocial responses to mortality salience, such as anxiety (Jonas et al., 2002), construal level, interdependent self-construal, global–local identity, or empathy. Testing these variables would provide a more comprehensive understanding of the psychological pathways linking mortality salience, identity, and prosocial behavior.

Third, our operationalization of prosocial behavior involved global causes, which may have accentuated the relevance of global identity. Future studies could investigate prosocial behaviors directed toward local causes (e.g., neighborhood initiatives) and include objective or behavioral measures of social connectedness (e.g., network size or contact frequency) to complement self-reported perceptions. While we followed prior literature in categorizing participants as relatively global or local identifiers, examining identity as a continuous construct may reveal further nuances.

Finally, our sample was relatively young (mean ages 25 and 31) and drawn solely from the United States, which may constrain the generalizability of the findings. Prior research suggests that both age and cultural background can moderate responses to mortality salience (e.g., Maxfield et al., 2007). Future research should examine whether the observed interaction effects between mortality salience and global–local identity on prosocial behavior extend to older or more culturally diverse populations.

## Data Availability

The data presented in this study are available on request from the corresponding author.
